# Unilateral Absence of Sternocleidomastoid and Ipsilateral Trapezius Presenting as Congenital Torticollis: A Case of a Rare Entity

**DOI:** 10.7759/cureus.17222

**Published:** 2021-08-16

**Authors:** Siddhi Chawla, Anupama Tandon, Gaurav Meena

**Affiliations:** 1 Department of Radiology, University College of Medical Sciences, Delhi, IND

**Keywords:** congenital torticollis, sternocleidomastoid, trapezius, cerebellar hypoplasia, agenesis

## Abstract

The most common cause of congenital torticollis is sternocleidomastoid contracture. Torticollis due to a unilateral absence of sternocleidomastoid is very rare. Association of an ipsilateral absence of sternocleidomastoid and trapezius with cerebellar hypoplasia is even rarer. We describe a combination of these rarities in an 11-year-old patient with congenital torticollis.

## Introduction

Congenital absence of skeletal muscle is rare and has an incidence of one in 11,000 births [1]. To date, there are only around a dozen reported cases presenting with congenital unilateral agenesis of sternocleidomastoid (SCM) muscle [2], with only a few of them with torticollis. Further, a combined absence of SCM and trapezius is exceedingly rare. Only three reported cases could be found even on extensive literature search [3,4]. Another reported case is that of absent SCM associated with cerebellar hypoplasia [5].

Differentiation of the commoner causes of congenital muscular torticollis (CMT) apart from muscular agenesis is essential as the treatment offered is different. SCM agenesis can be associated with lung herniation into the neck, a sinister condition requiring surgical correction. We report a case of an 11-year-old patient who had a combination of rare associations, i.e., agenesis of right sternocleidomastoid and trapezius with ipsilateral cerebellar hypoplasia presenting with torticollis.

## Case presentation

An 11-year-old boy had presented to the orthopedic department with the complaint of head tilt to the left along with facial deformity in the form of a small left side of the face and skull. He had a full-term normal vaginal delivery in hospital and was apparently normal at birth. The parents noticed a head tilt to the left when the boy was six months of age but they did not seek any medical help at that time. The condition progressed mildly over the years, however, no physical discomfort was experienced by the patient. The child had adjusted well to this tilt. There was no history of similar complaints in other family members.

**Figure 1:**
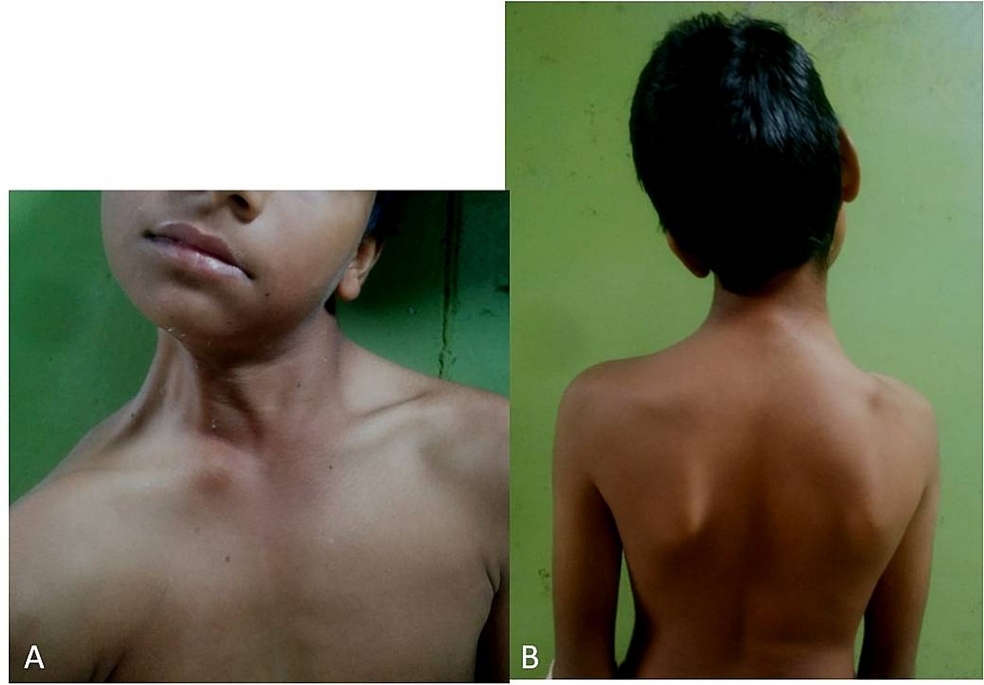
A. Eleven-year-old patient with the head tiltings to left and the chin towards the opposite shoulder. The left sternocleidomastoid is prominent. There is no muscle bulge for the right sternocleidomastoid. Lateral neck muscles are prominent on the right side. B. The right scapula is prominent.

On inspection, we noticed an asymmetry in the contour of the neck, the shoulder, and the bilateral scapula. Anterior neck muscles were prominent on the left with the the head turned to the left side (Figure 1A). The left shoulder was at a higher level than the right, the left scapula was elevated and there was mild scoliosis to the left (Figure 1B). The patient had no restriction in flexion and extension movements in the anteroposterior (AP) direction or tilting the neck to the left side, however, there was a significant restriction in tilting the head to the right side. In view of the above findings, a clinical diagnosis of contracture of left SCM was made and the patient was referred for imaging.

**Figure 2:**
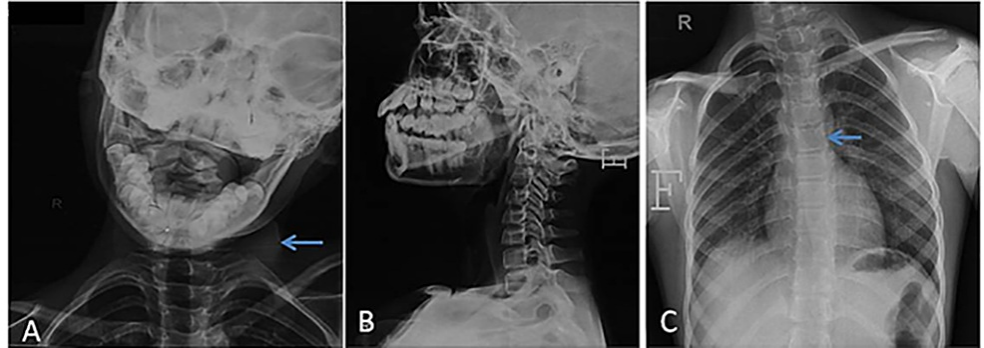
A. X-ray AP spine shows soft tissue bulge on the left side (blue arrow). B. Lateral X-ray of the cervical spine and C. X-ray chest were done to rule out vertebral anomalies. It showed mild scoliosis to the left (blue arrow). No vertebral/rib/spine structural abnormalities were seen. AP: anteroposterior.

**Figure 3:**
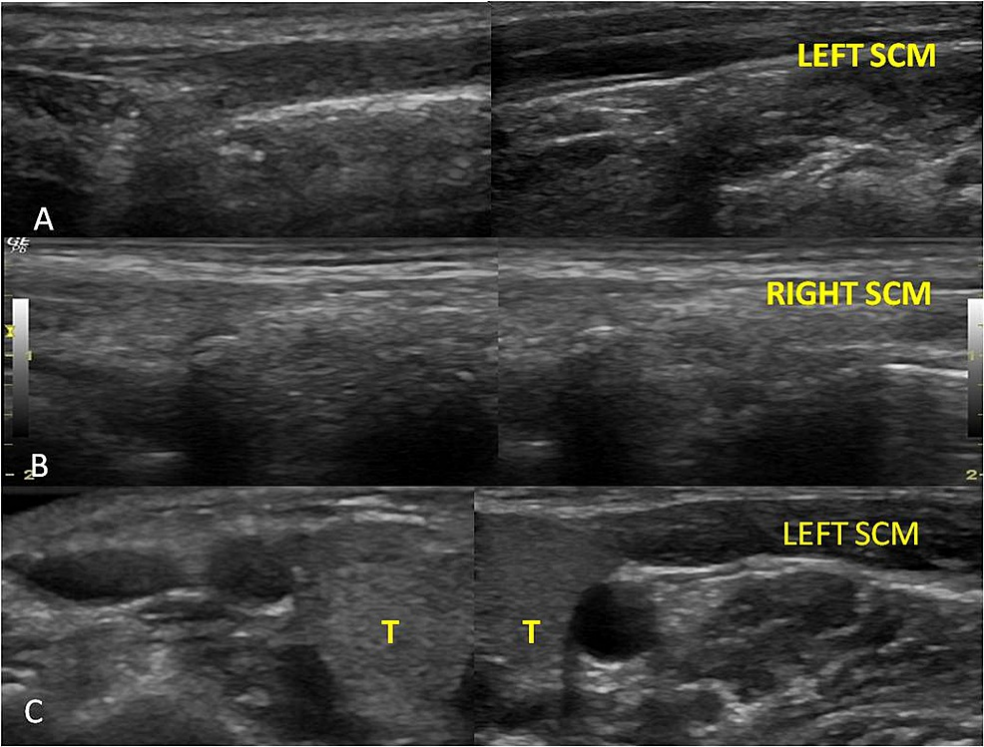
A. Longitudinal view shows the normal left sternocleidomastoid in the entire length. B. Longitudinal view shows the absent right sternocleidomastoid. C. Axial view at the level of thyroid shows the absent sternocleidomastoid on the right side and normal sternocleidomastoid on the left side T: thyroid gland, SCM: Sternocleidomastoid.

**Figure 4:**
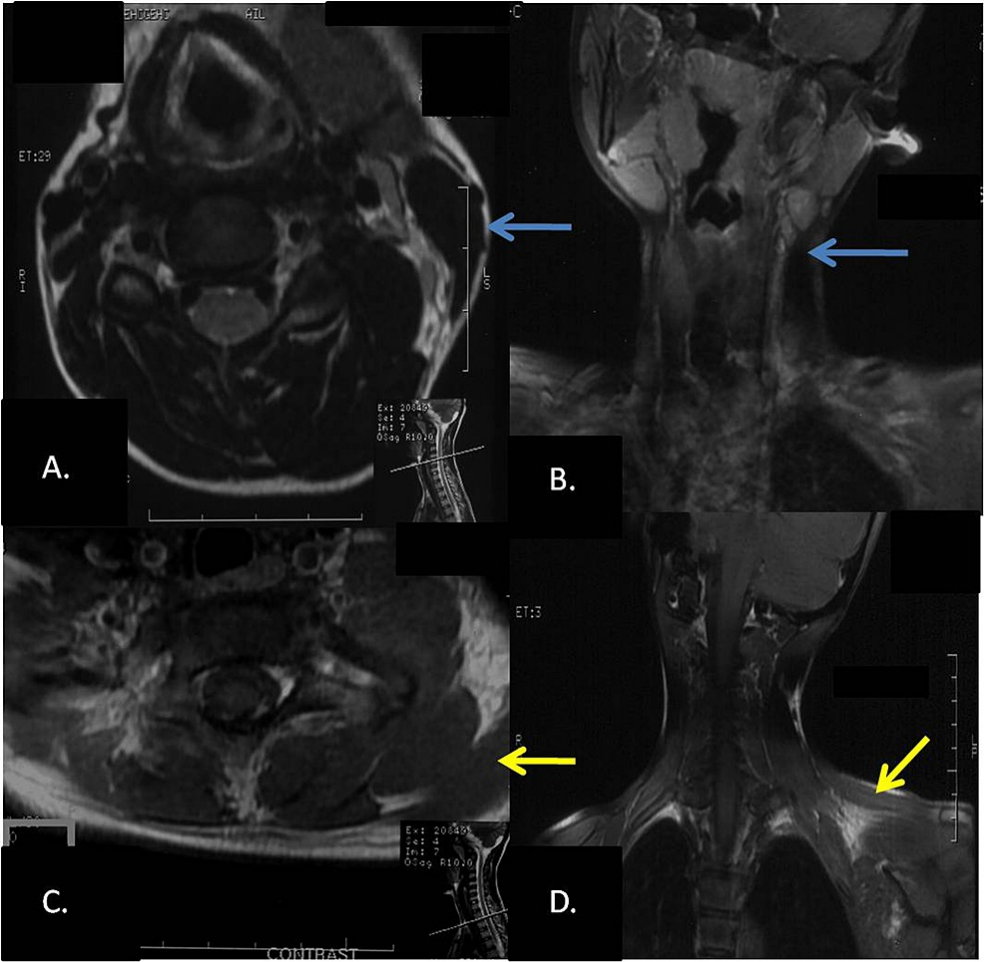
A. Axial T2-weighted image and B. coronal T1-weighted image show the normal left sternocleidomastoid (blue arrow) and absent on the right SCM. C. Post-contrast T1-weighted axial image and D. coronal image show the normal trapezius on the left side (yellow arrow), which is absent on the right side. SCM: sternocleidomastoid; panel A: inset shows the level of axial section at C4-C5 intervertebral disc level; panel C: inset shows the level of axial section at T1-T2 intervertebral disc level.

**Figure 5:**
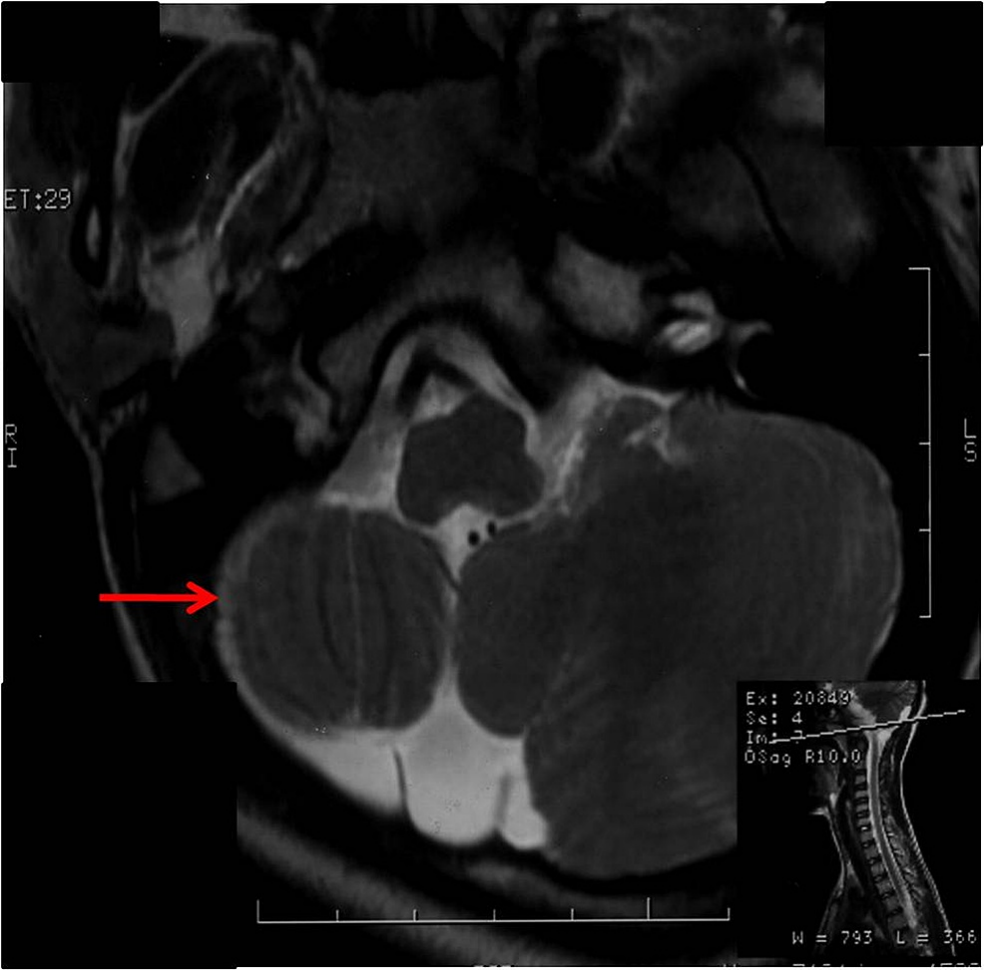
Axial T2-weighted images show the hypoplastic right cerebellar hemisphere. Inset shows the axial section taken at the level of the basisphenoid.

Due to asymmetry in the shoulder position, a radiograph of the neck (Figure 2A) was acquired to look for any bony anomalies. The radiograph of the neck (Figure 2B) and the chest (Figure 2C) were largely unremarkable and all the vertebrae and bilateral shoulder joints were normal. However, mild scoliosis was seen. Ultrasound (US) of the neck region was subsequently performed (Figure 3). On examining the patient with a linear probe, the right sternocleidomastoid muscle was not visualized and was replaced by a fibrotic hypoechoic structure seen extending from the mastoid process to the clavicle. The left SCM, however, was normal in size and echotexture. No focal lesion was seen within it. A provisional diagnosis of atretic or aplastic sternocleidomastoid was made. Subsequent evaluation with contrast-enhanced MRI of the neck demonstrated the complete absence of the right SCM muscle along with the absence of the ipsilateral trapezius muscle (Figure 4A-4D). The left sternocleidomastoid was normal. The sections of the posterior fossa revealed hypoplasia of the right cerebellar hemisphere (Figure 5). The spine, however, was normal. The right lung was normal in position and no herniation into the neck was seen. At present, the patient had marked torticollis as well as cosmetic deformity and was thus advised release.

## Discussion

Torticollis describes an abnormal neck posture with the lateral translation of head on trunk, variable degrees of lateral head tilt, and neck rotation. It can be either congenital or acquired in nature [3]. Depending upon the age and the duration of symptoms the differential diagnosis for torticollis differs drastically for infants, children, and adolescents. In the pediatric age group, CMT is the most common cause of torticollis in infants. It is also known as fibromatosis colli or pseudotumor of infancy. Other congenital causes include vertebral anomalies like Klippel- Feil syndrome, ocular torticollis due to the weakness of one of the extraocular muscles, Sandifer syndrome (abnormal intermittent gastroesophageal reflux with or without hiatus hernia), neoplastic lesions like posterior fossa tumors, and benign paroxysmal torticollis of infancy [6]. Congenital absence of the sternocleidomastoid muscle also has been reported as a rare but known cause of torticollis. Although the pathogenesis is not known but various factors have been implicated like inflammatory, vascular, neuropathic, or myopathic insult during embryogenesis [7] and rarely a genetic cause [8]. McKinley and Hamilton [7] described the torticollis secondary to the absence of SCM as a dynamic deformity. Due to unopposed action of the normal SCM muscle leading to postural deformity in the form of head tilt away from the affected side and chin to point towards the shoulder of the affected side. However, if neglected, in early infancy, due to constant postural tilt, the child develops permanent facial deformity, which was also seen in our case. In few cases, the lung herniation into the neck associated with a unilateral congenital absence of the sternocleidomastoid muscle has been reported [9]. As the herniated the lung has the potential to undergo incarceration and is along with neurovascular structures in the neck is susceptible to trauma, hence, corrective surgery is usually undertaken. In the above case, there is an association of unilateral absence of SCM with the ipsilateral absent trapezius muscle. There was no associated ipsilateral lung herniation as reported in the literature in cases with a similar presentation [5].

The association of unilateral congenital agenesis of sternocleidomastoid with trapezius muscles is known and was earlier reported usually via cadaver dissections [2] and now verified by case reports. It is accompanied by the loss of innervations by the spinal accessory nerve and arterial supply. In one of the cases with complete congenital unilateral absence of trapezius [10], the ipsilateral arterial blood supply, the trapezius part of spinal accessory nerve, and dorsal scapular vessels were absent. There is, however, no restriction in the movements of the shoulder joint because of the synergistic action of rhomboids and levator scapulae, which compensate for the absence of SCM and trapezius [2].

In our case, the agenesis of sternocleidomastoid and trapezius was associated with ipsilateral cerebellar hypoplasia. There is only one case report on the association of sternocleidomastoid agenesis with ipsilateral cerebellar hypoplasia [5]. However, it was not associated with the agenesis of the trapezius. Unilateral cerebellar hypoplasia in itself is a rare condition and identified by the loss of volume in the cerebellar hemispheres. It can present clinically with varied severity and if acquired prenatally due to ischemic or vascular causes can cause severe developmental delay, speech delay, seizures, microcephaly, hypotonia, ataxia and impaired coordination, and abnormal movements (tremor); hypertonia, autistic features, ocular signs such as, nystagmus, strabismus, and abnormal ocular movements [11,12]. The child in our case, except for the cosmetic deformity due to delayed presentation, was neurologically sound and developmentally normal.

In any case of pediatric torticollis, one of the most important conditions that needs to be ruled out before considering the diagnosis of sternocleidomastoid agenesis is CMT. It is characterized by fibrosis of the sternocleidomastoid muscle due to an insult in the prenatal life or during birth [4]. US is one of the best screening modalities that can be used to diagnose this condition and also to differentiate CMT from agenesis. While CMT presents as a fibrotic hypoechoic mass within the muscle with unilateral contracture of the SCM, in congenital agenesis, there is complete absence of the SCM of the affected side when compared to the contralateral normal side. It is to be emphasized that strict attention should be given to symmetrical structures in the neck. It has been mentioned in previous studies that the remaining SCM muscle may be hypertrophied and can be misinterpreted as CMT, access to which may be limited due to the postural deformity of the patient as evident in our case due to late presentation. These limiting factors of ultrasound imaging can be overcome by the use of CT or MRI imaging. Since most the cases present in the pediatric age group, CT imaging is deferred as it involves radiation and there is poor contrast resolution apart from the cases with suspected lung herniation. Thus, MRI is the modality of choice in young patients. It allows thorough examination of the musculature as well as to rule out other causes of torticollis. Children suspected of agenesis of sternocleidomastoid on initial ultrasound scan can undergo MRI to confirm the diagnosis and provide additional associated findings like ipsilateral agenesis of the trapezius and cerebellar hypoplasia as seen in our case. It is necessary to differentiate the causes of torticollis as the treatment in children has to be started as early as possible. Persistent torticollis beyond one year of age does not respond well with only conservative therapy and the need for corrective surgery such as the release of the fibrotic band increases [3]. The cosmetic deformity is corrected surgically with customized dermal grafts or fasciocutaneous flaps with fascial extensions like an inframammary, extended circumflex scapular flap can be used [9]. Frost et al. [2] performed a release surgery in a case of unilateral absence of SCM with encouraging results. Authors have also reported that uncorrected torticollis can result in complaints like movement limitation, facial asymmetry and diplopia as seen in our patient. Although surgical management has been beneficial in improving the degree of torticollis in adults with neglected congenital muscular torticollis, facial asymmetry and diplopia still persist even after the surgery. These authors thus explained the need for early diagnosis and treatment to provide better results [13]. Most cases with unilateral SCM absence either had no torticollis or only mild impairment, which improved on physical therapy. However, our patient had severe torticollis and would have benefitted from surgery. Unfortunately, the parents did not consent to surgery.

## Conclusions

Despite its rarity, the unilateral absence of the SCM should be considered as a cause of torticollis in infants and in young patients with facial deformity and with congenital torticollis. The associated conditions such as ipsilateral absent trapezius, cerebellar hypoplasia, and lung herniation should also be sought for keeping in mind the other differentials and imaging pitfalls.
